# The state of enrollment on the National Health Insurance Scheme in rural Ghana after eight years of implementation

**DOI:** 10.1186/s12939-019-1113-0

**Published:** 2019-12-31

**Authors:** Anthony Kwarteng, James Akazili, Paul Welaga, Philip Ayizem Dalinjong, Kwaku Poku Asante, Doris Sarpong, Samuelina Arthur, Martin Bangha, Jane Goudge, Osman Sankoh

**Affiliations:** 1 Kintampo Health Research Center, Ghana Health Service, P. O. Box 200, Kintampo, Ghana; 2 Navrongo Health Research Center, Ghana Health Service, Navrongo, Ghana; 3 Dodowa Health Research Center, Ghana Health Service, Dodowa, Ghana; 4 INDEPTH Network, Accra, Ghana; 5 Center for Health Policy/MRC Health Policy Research Group, School of Public Health, Faculty of Health Sciences, University of the Witwatersrand, Johannesburg, South Africa

**Keywords:** National health insurance scheme, Universal health coverage, Exemption policy, Wealth index, Poor, Equity, Ghana

## Abstract

**Background:**

In 2004, Ghana implemented a national health insurance scheme (NHIS) as a step towards achieving universal health coverage. In this paper, we assessed the level of enrollment and factors associated with NHIS membership in two predominantly rural districts of northern Ghana after eight years of implementation, with focus on the poor and vulnerable populations.

**Methods:**

A cross-sectional survey was conducted from July 2012 to December 2012 among 11,175 randomly sampled households with their heads as respondents. Information on NHIS status, category of membership and socio-demographic characteristics of household members was obtained using a structured questionnaire. Principal component analysis was used to compute wealth index from household assets as estimates of socio-economic status (SES). The factors associated with NHIS enrollment were assessed using logistic regression models. The reasons behind enrollment decisions of each household member were further investigated against their SES.

**Results:**

Approximately half of the sampled population of 39,262 were registered with a valid NHIS card; 53.2% of these were through voluntary subscriptions by payment of premium whilst the remaining (46.8%) comprising of children below the ages of 18 years, elderly 70 years and above, pregnant women and formal sector workers were exempt from premium payment. Despite an exemption policy to ameliorate the poor and vulnerable households against catastrophic health care expenditures, only 0.5% of NHIS membership representing 1.2% of total exemptions granted on accounts of poverty and other social vulnerabilities was applied for the poor. Yet, cost of premium was the main barrier to NHIS registration (92.6%) and non-renewal (78.8%), with members of the lowest SES being worst affected. Children below the ages of 18 years, females, urban residents and those with higher education and SES were significantly more likely to be enrolled with the scheme.

**Conclusions:**

Despite the introduction of policy exemptions as an equity measure, the poorest of the poor were rarely identified for exemption. The government must urgently resource the Department of Social Welfare to identify the poor for NHIS enrollment.

## Introduction

Universal health coverage (UHC) through a pre-payment financing mechanism remains a key strategy in achieving the sustainable development goals (SDG) [1]. In 2004, Ghana was among few low- and middle-income countries (LMIC) in Africa and Asia that implemented a national health insurance scheme (NHIS) as a step towards achieving UHC [2].

Prior to implementation of the NHIS in Ghana, the health financing regime was dominated by the user fees introduced by the World Bank in the 1980s [3, 4]. The infamous user fee policy known as the “cash and carry system” required direct payment of health services at the point of care [5, 6]. There is ample evidence to demonstrate that the user fee policy resulted in catastrophic health care expenditures and reduced access to health care services particularly for the poor and vulnerable households [7–10]. The introduction of the NHIS was therefore intended to pool risks and financial resources, and remove financial barriers for equitable access to health care for all citizens especially the poor and vulnerable [11–13]. The policy objective of the scheme as set out during its introduction in 2003 was to ensure within five years, universal enrollment for all residents in Ghana [14, 15].

However, available data suggest far lower level of NHIS coverage than has been envisaged, with disproportionately low representation of the poor and vulnerable. In a study conducted in the Asante Akim North District of the Ashanti Region in 2008, only 38% of the residents were found to be registered with households of the lowest SES at 4.9 times significantly less likely to be registered compared to those of the highest SES [16]. The computation of NHIS enrollment based on yearly cumulative subscriptions rather than active membership has been critiqued by many including civil society organizations as inaccurate [17]. This approach resulted in over-estimation of NHIS coverage as it failed to rightly adjust for persons who may have either registered twice, in possession of an expired NHIS card, dead or migrated. Despite downward review of NHIA (National Health Insurance Authority) previously estimated coverages of 53–60% of the population including the enrollment figure of 2010 to 33% as a way of correcting this anomaly, Oxfam, an international non-governmental organization reported in 2011 that only 18% of the population resident in Ghana were registered with the scheme [17, 18].

In this study, we assessed the level of enrollment and factors associated with NHIS membership in two predominantly rural districts of northern Ghana, with focus on the poor and vulnerable populations. We further assessed willingness for future enrollments and the factors influencing these decisions. Findings from our study will inform decision-makers on the level of NHIS coverage and factors that influence membership for effective planning and equitable inclusion of vulnerable populations.

This study forms part of a comparative assessment of the effect of health care financing reforms undertaken by two LMIC (Ghana and Vietnam) in the same era with the goal of achieving UHC. The study leveraged on the existence of health and demographic surveillance systems (HDSS) at two predominantly rural settings; one in the Kassena-Nankana districts of Ghana (Africa) and the other, Filabavi Province of Vietnam (Asia) to evaluate these reforms.

### Overview of the Ghana NHIS

The NHIS was established by the NHIS Act, 2003 (Act 650) with the aim of removing financial barriers to health care services for all residents in Ghana. Until the enactment of the new NHIS Act, 2012 (Act 852) that established the NHIA, the scheme operated semi-autonomous district-wide (public) mutual health insurance schemes (DMHIS) [19]. Under the current regime, the operations of the erstwhile 155 individual DMHIS have been harmonized under the NHIA to ensure effective management for efficient service delivery.

The NHIA has a pre-defined benefit package of about 95% of the most common diseases seen at health care facilities in Ghana. The services covered include general out-patient and in-patient care, reproductive and maternal care (normal and caesarean delivery), eye, dental and emergency care. A list of essential drugs pre-qualified by the NHIA is also covered. However, expensive procedures such as dialysis for chronic renal failure, treatments for cancer (other than cervical and breast cancers), organ transplants and cosmetic surgery are not covered. Treatment for HIV/AIDS (Human Immuno-deficiency Virus/Acquired Immune Deficiency Syndrome), immunization and family planning services are also not covered under the scheme but provided for under other government vertical programs.

The scheme has a cocktail of funding sources with tax constituting the majority of about 90% of total annual inflows. The funding source comprises of a 2.5% value added tax levied on selected goods and services consumed in the country, 2.5% of workers’ SSNIT (Social Security and National Insurance Trust) pension fund deductible at source, annual premium and registration fees. Others are returns on investments and support from international donors.

Ghana’s NHIS card is valid for 5 years but subject to annual renewals either by payment of premiums and/or registration fees. By default, all SSNIT contributors are exempt from paying premium but required to pay a registration fee for NHIS card in order to access health care services under the scheme. Voluntary subscribers from the informal sector are required to pay income-adjusted yearly premium in addition to a registration fee. However, in practice, a flat premium is charged by many DMHIS due to the difficulty in assessing household income levels. The annual premium for residents in the Kassena-Nankana East and Kassena-Nankana West districts from January to December 2012 was fixed between GH¢7.20 and GH¢47.70 (US$8.00–$53.00) whilst registration was GH¢2.00 (US $2.20).

To promote access to child, reproductive and maternal health services for the attainment of the then millennium development goals, pregnant women and children below the ages of 18 years were exempt from paying premium [20]. Other exempts groups are the elderly aged 70+ years and above, and individuals considered indigent. Apart from pregnant women who have to show evidence of their pregnancy for a NHIS card, the < 18-year-olds and elderly 70+ years are however required to pay a registration fee.

The NHIA introduced capitation as alternative payment mechanism to contain the escalating cost of claims by sharing financial risks among the scheme, providers and subscribers. It was piloted in the Ashanti Region in 2012. At the time of this study, capitation was not operational in the Upper East Region.

## Methods

### Study setting

**Fig. 1:**
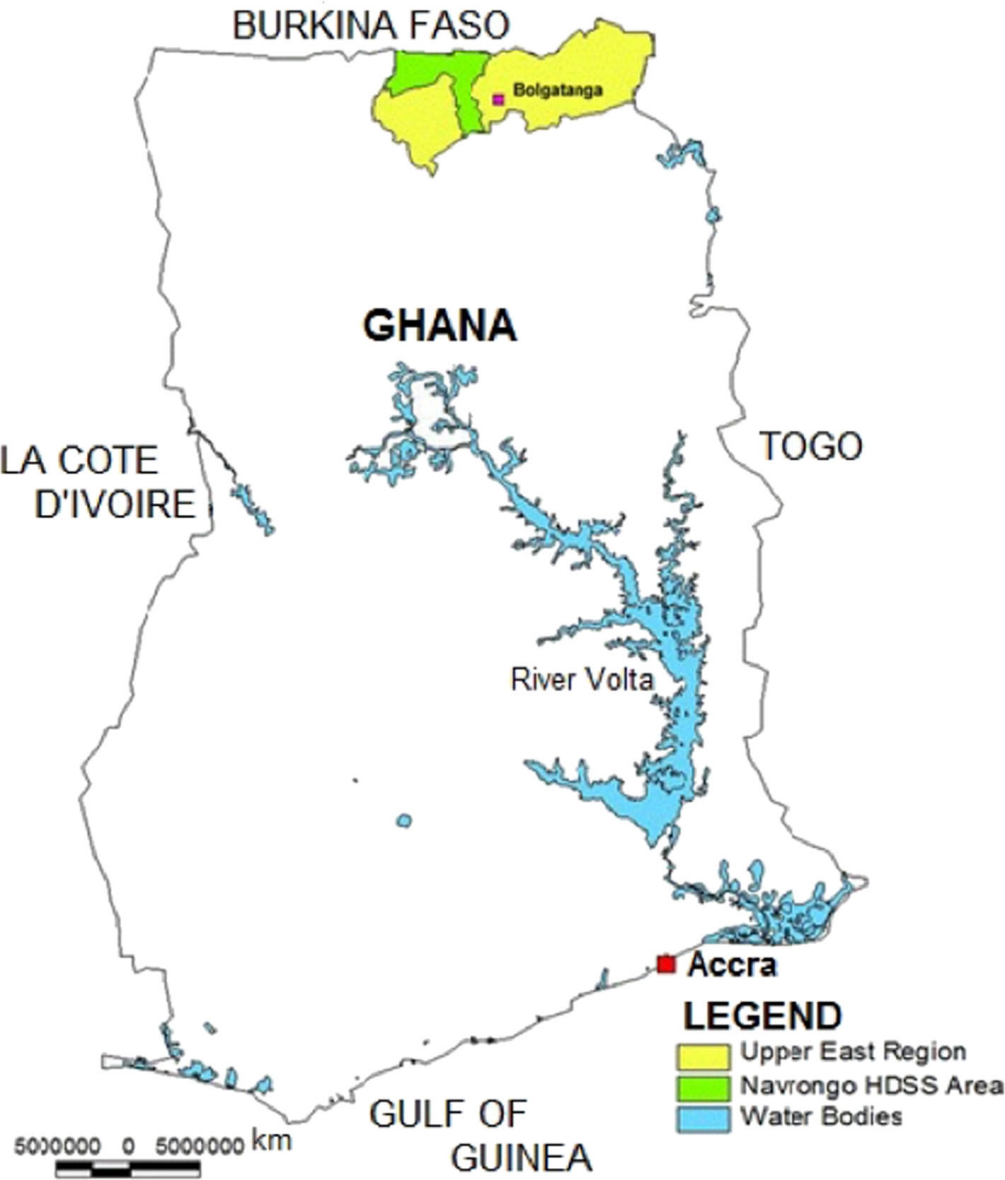
Map of Ghana showing the study area

The study was conducted in the Kassena-Nankana East and Kassena-Nankana West, two adjoining districts of the Upper East Region of northern Ghana. They cover an area of approximately 1675 km^2^ (Fig. 1).

The districts have a resident population of about 152,000 living in 32,000 households [21]. The Kassenas and Nankanis are the dominant ethnic groups. Of those employed 15 years and older, 95.2% worked in the informal sector whilst only 4.6% in the formal sector [22]. A majority of the population are subsistence farmers engaged mainly in the cultivation of millet, sorghum and rice; and rearing of cattle. Poverty indicators are far worse than the national average [23]. The mean annual household expenditure and per capita expenditure of the region according to report of the Ghana Living Standard Survey in 2008 was GH¢1066.00 (US $888.30) and GH¢229.00 (US $190.80) compared to the national averages of GH¢1918.00 (US $1598.30) and GH¢644.00 (US $536.70) respectively.

Like other parts of Ghana, malaria is the most frequently diagnosed disease and managed at the community and/or primary health care levels [24].

There are 8 health centers, 3 private clinics; and one hospital that serves as the district referral hospital. At the community level, 27 community-based health planning services popularly known as CHPS compounds are manned by resident community health officers who also provide door-to-door health services to the people.

The two districts are under continuous surveillance by the Navrongo Health and Demographic Surveillance System (NHDSS) that tracks population changes, assess the burden of diseases and evaluate policy interventions. Each resident is assigned a unique permanent identification (Perm ID) and visited 3 to 4 times a year for updates on key health and demographic indicators [21]. During these visits, information on ownership of household assets is obtained.

### Training and pretesting for data collection

The data collectors comprised of field workers and research assistants who had a minimum of three (3) years field data collection experience. They were trained for four (4) weeks on the study before field data collection began. The training began with the introduction of the study objectives, design and procedures in a classroom setting. The role of the interviewers in the survey and techniques of conducting interviews in order to solicit the relevant information were discussed. Training manuals on these topics were used. Answers to questions were explained to enable trainees have firm understanding of the questionnaire.

The third week of training focused on mock interviews in the local languages during which trainees were supervised to conduct trial interviews among themselves. This was followed by a pilot of the survey activities outside the study area. The suitability of the study questionnaires and other field challenges encountered during the pilot were discussed and addressed before the survey commenced.

### Sampling and data collection

The listing of households from the database of NHDSS was used as the sampling frame. Using a written program in STATA, version 12 (StataCorp, College Station, Texas, United States), 12,000 households were randomly sampled. The Perm ID of each member of the sampled households along with their first names was extracted.

The selected households were visited by trained field workers who conducted the interviews. The interviews were started in July 2012 and ended in December 2012. The respondents were heads of households. A household was defined as a person or group of persons who lived together in the same house or compound and shared the same house-keeping arrangements [25]. The head of a household was a member of that household who bears the economic and social responsibilities for the household and is recognized as such by other members. In the absence of a household head, an older representative who takes responsibility for the upkeep of that household and has information on the NHIS status of all household members was deemed eligible for the interview. Other members of the household were allowed to assist the main respondent in answering the questionnaire. A sampled household without an eligible respondent on three visits within five (5) working days were left out for further interview. All field activities were supervised by experienced trained research officers who have a minimum of basic degree in the social sciences.

The interviews were administered by the use of structured questionnaires after informed written consents have been obtained. Using individual perm ID, the background characteristics of household members, their health insurance status, and category of membership (if registered with the scheme) were recorded. To ensure validity of health insurance status, the expiry date of NHIS card of each household member was inspected. Any recent history of illness or injury of a household member in one calendar month preceding the date of interview was recorded.

In addition, the willingness to renew NHIS membership on expiration and the reasons for the decision were solicited. Similarly, the reasons for non-registration for those who had previously been insured or never registered were also investigated. The factors explored included affordability of insurance premium, convenience of access to health care services, adequacy of the benefit package, previous benefits received or access to health care services in the event of ill-health without any financial burden. Other factors were availability of drugs, exemption from premium payment, waiting time, NHIS card processing time, attitude of health workers and insurance staff.

The Institutional Ethics Committee of the Navrongo Health Research Center granted ethical approval for the study. All data were kept confidential with restricted access.

### Data management and statistical analysis

All data forms were manually checked for completeness and consistency before data processing began. The data were independently entered by two clerks and validated in FoxPro 6.0.

The analysis was done in STATA, version 12 (StataCorp, College Station, Texas, United States) after further cleaning among the variables have been performed. Statistical point estimates were computed and presented as means, proportions or percentages for the background characteristics and NHIS membership.

The household wealth index was computed using principal component analysis from household assets obtained during the routine HDSS visits. The asset list is comprised of more than twenty-five (25) items that range from smaller household items (e.g. radio, fan, wall clocks) to expensive assets (e.g. vehicle, landed properties, cow). The households were then categorized into five socio-economic bands namely poorest, moderately poor, average, moderately rich and richest; and made to reflect each member of the respective household. Using individual perm ID, the estimated wealth index of each household member was linked to his/her survey data.

Bivariate analysis was employed to identify socio-demographic factors that were possible predictors of NHIS membership. Identified variables with level of significance (*p* < 0.05) were modelled into the multivariate logistic regression and adjusted odds ratios with 95% confidence intervals were calculated. Reference categories were defined as those usually associated with the least NHIS registration rates.

The category of NHIS membership was assessed relative to socio-demographic factors. Data on the recent history of illness was used as a proxy to assess need for health care services and therefore uptake of insurance. The reason influencing one’s enrollment decision including future retention relative to his/her SES was presented as bar charts.

### Definition of key study variables

In our analysis, one key variable was the NHIS status of individual household members. An individual was considered insured if a valid NHIS card was seen by the data collectors. Holders of expired health insurance card were classified as “previously insured” whilst those who reported never to have registered on the scheme were described as “never insured”. The membership of NHIS was categorized into the formal sector, informal sector and the exempt groups. The formal sector comprised of employees including those self-employed who contribute to the workers’ SSNIT pension fund. They are mostly teachers, health workers, civil servants and police officers. Other public employees such as orderlies, security agents, messengers and cooks also belong to the formal sector. The informal sector on the other hand is made up of non-contributors of workers’ SSNIT pension fund who voluntarily subscribe to the scheme through payment of annual premium. They are mostly farmers, artisans and traders. The exempt groups are free from premium payments and consist of two categories. The exempt (children, elderly and pregnant women) or “exempt CEP” is made up of children below the ages of 18 years, the elderly aged 70 years and above, and pregnant women. The other, “exempt poor” are the indigents who do not have any gainful employment or income, fixed residence or identifiable support from other persons.

## Results

### Description of the sampled population

A household response rate of 93.1% (11,175/12,000), involving data from a total of 55,992 members was recorded over 6 months of data collection. For the purposes of our analysis, we excluded 16,730 individuals from 1599 sampled households whose NHIS cards could not be verified. Our analysis therefore involves data on 39,262 individuals, successfully linked to their corresponding socio-economic information from 9576 households.

### Background characteristics of individual household members

The socio-demographic characteristics of the sampled population are presented in Table 1. The sample was a productive age population with working-age adults 18–59 years constituting the majority of 46.7% followed by the < 18-year-olds (41.6%). There were more females than males (51.7% vs. 48.3%). Most residents (89.3%) lived in the rural areas and in households of at least five members (75.5%). The Kassenas constituted 51.5% of the sampled population and the Nankanis, 44.2%.

**Table 1:** Socio-demographic characteristics of household members by insurance status (%). *N* = 39,262

Characteristic	Categories	Sub-total		Currently insured	Previously insured	Never insured
		n	%			
Age (years)	< 18	16,328	41.6	53.2	17.2	29.6
	18–59	18,352	46.7	48.1	22.1	29.8
	60–69	2682	6.8	46.9	21.6	31.5
	70+	1900	4.8	46.2	23.4	30.4
Gender	Female	20,318	51.7	53.5	20.3	26.2
	Male	18,944	48.3	46.3	19.8	33.9
Location	Rural	35,077	89.3	46.7	20.4	32.9
	Urban	4185	10.7	77.8	17.1	5.1
Household size	1	869	2.2	53.9	21.2	25.0
	2–4	8740	22.3	52.8	18.0	29.2
	5+	29,653	75.5	49.1	20.7	30.2
Education	No formal	10,321	26.3	42.8	21.5	35.6
	Primary	12,946	33.0	45.3	20.8	33.9
	High school	7450	19.0	56.2	22.2	21.7
	Tertiary	889	2.3	77.6	14.9	7.5
	Under schooling age*	7656	19.5	58.5	15.6	25.9
Ethnicity	Kassena	20,216	51.5	61.3	19.4	19.3
	Nankani	17,350	44.2	35.1	21.0	43.9
	Others	1696	4.3	68.4	18.6	13.0
Religion	Christianity	19,704	50.2	59.0	19.5	21.5
	Traditional	17,556	44.7	37.9	20.9	41.2
	Islam	2002	5.1	67.6	18.8	13.6
Wealth quintiles	Poorest	11,106	28.3	41.7	20.5	37.9
	Moderately poor	8765	22.3	42.5	20.8	36.7
	Average	7784	19.8	44.2	23.0	32.8
	Moderately rich	7403	18.9	60.3	18.5	21.3
	Richest	4204	10.7	80.5	15.2	4.4
Recent illness	Yes	2172	5.5	53.2	17.1	29.7
	No	37,090	94.5	49.8	20.3	29.9
Total		39,262	100.00	50.0	20.1	29.9

*Represents children yet to attain the schooling age of 6 years

Over a quarter of the sampled population had no formal education (26.3%), one-third with primary education whilst only 2.3% had tertiary education. The majority were from households of the lowest wealth quintiles (28.3%). Only 5.5% of the people had reported ill or injured in the month preceding the survey.

### Insurance status by socio-demographic characteristics

The enrollment status of household members by socio-demographic characteristics is summarized in Table 1. Of the sampled population of 39,262 household members, approximately half (50.0%) were currently registered; 20.1% had previously been registered whilst 29.9% had never registered with the scheme.

The < 18-year-olds had the highest proportion of current registration (53.2%) followed by the working-age adults 18–59 years (48.1%) whilst the elderly 70+ years had the least registration of 46.2%. More females than males were registered (53.5% vs. 46.3%). A higher proportion of the sample was registered from the urban settings (77.8%) than rural areas (46.7%). Individuals with the highest education recorded the highest registration of 77.6% whilst those with no education had the least registration of 42.8%. With respect to SES, members of the richest households had the highest proportion of NHIS registration (80.5%) compared to the least registration of 41.7% from the poorest households.

Non-renewal of NHIS membership was mostly common among the elderly aged 70+ years (23.4%), household members of average SES (23.0%) and high school graduates (22.2%).

Among members who have never joined the scheme, the Nankanis (43.9%), traditional worshippers (41.2%), those of the poorest households (37.9%), without formal education (35.6%) and males (33.9%) were in the majority. However, fewer than 8.0% of those of the richest households (4.4%), urban residents (5.1%) and tertiary graduates (7.0%) have never joined the scheme.

### Factors associated with NHIS enrollment

Table 2 presents the factors associated with NHIS enrollment in the bivariate and multivariate analyses. In the bivariate analysis, being a child, female, urban resident, from households of at least five members, either a Christian or Muslim was significantly associated with NHIS membership. Other significant predictors were belonging to the Kassena ethnic group, formally educated, of at least average SES and having experienced no recent illness.

**Table 2:** Factors associated with NHIS membership in the Kassena-Nankana East and Kassena-Nankana West districts in 2012, *N* = 39,262

Factors	Categories	% currently insured	Odds ratio (95% Confidence Interval)	
			Unadjusted	Adjusted
Age (years)	< 18	53.2	1.3 (1.2–1.5)	1.2 (1.1–1.4)^***s***^
	18–59	48.1	1.1 (1.0–1.2)	0.8 (0.8–0.9)
	60–69	46.9	1.0 (0.9–1.2)	1.0 (0.9–1.1)
	70+	46.2	1.0 (base)	1.0 (base)
Gender	Female	53.5	1.3 (1.3–1.4)	1.4 (1.3–1.5)^***s***^
	Male	46.3	1.0 (base)	1.0 (base)
Location	Rural	46.7	1.0 (base)	1.0 (base)
	Urban	77.8	4.0 (3.7–4.3)	1.4 (1.3–1.5)^***s***^
Household size	1	53.9	1.0 (base)	1.0 (base)
	2–4	52.8	1.0 (0.8–1.1)	1.0 (0.9–1.2)
	5+	49.1	0.8 (0.7–1.0)	1.0 (0.9–1.2) ^***n***^
Education	No formal	42.8	1.0 (base)	1.0 (base)
	Primary	45.3	1.1 (1.1–1.2)	1.0 (0.9–1.0)^***n***^
	High School	56.2	1.7 (1.6–1.8)	1.4 (1.3–1.5)^***s***^
	Tertiary	77.6	4.6 (3.9–5.4)	2.4 (2.0–2.9)^***s***^
	Under schooling age*	58.5	1.9 (1.8–2.0)	1.4 (1.3–1.5)^***s***^
Ethnicity	Kassena	61.3	2.9 (2.8–3.1)	2.3 (2.2–2.4)^***s***^
	Nankani	35.1	1.0 (base)	1.0 (base)
	Others	68.4	4.0 (3.6–4.5)	2.6 (2.3–3.0)^***s***^
Religion	Christianity	59.0	2.4 (2.3–2.5)	1.5 (1.5–1.6)^***s***^
	Traditional	37.9	1.0 (base)	1.0 (base)
	Islam	67.6	3.4 (3.1–3.8)	1.2 (1.1–1.4)^***s***^
Wealth quintiles	Poorest	41.7	1.0 (base)	1.0 (base)
	Moderately poor	42.5	1.0 (1.0–1.1)	1.0 (1.0–1.1)
	Average	44.2	1.1 (1.1–1.2)	1.1 (1.0–1.1)^***n***^
	Moderately rich	60.3	2.1 (2.0–2.3)	1.8 (1.7–1.9)^***s***^
	Richest	80.5	5.8 (5.3–6.3)	2.9 (2.6–3.2)^***s***^
Recent illness	Yes	53.2	1.0 (base)	1.0 (base)
	No	49.8	0.9 (0.8–1.0)	0.8 (0.7–0.9)^***s***^

^*^Represents children yet to attain the school-going age of 6 years

^s^Significant in both bivariate and multivariate analyses

^n^Not significant in the multivariate analysis

Members of the richest households had 5.8-fold increased odds of being registered on the scheme compared to those of the poorest households. Tertiary graduates had 4.6-fold increased odds of being registered compared to those without any formal education. Urban residents had fourfold increased odds of being registered compared to their rural counterparts. Muslims had 3.4-fold increased odds of being registered compared to traditional worshippers. The Kassenas were associated with nearly threefold increased odds of NHIS membership compared to the Nankanis. The < 18-year-olds were associated with 1.3-fold increased odds of NHIS membership compared to the elderly aged 70+ years. Females were likewise associated with 1.3-fold increased odds of being registered compared to their male counterparts.

In the multivariate analysis, all factors found to be significant in the bivariate analysis remained important predictors of NHIS membership, except for membership to households of at least five persons, being of average SES or having a primary education only. The factors conferring the strongest odds of NHIS membership were SES, ethnicity, education, religion, location of residence and age.

Members of the richest households were 2.9 times (AOR [adjusted odds ratio] = 2.9, 95% CI: 2.6–3.2) more likely to be registered with the scheme compared to those of the poorest households. Compared to the Nankanis, the Kassenas and people of other ethnic groups were respectively associated with 2.3-fold- (AOR = 2.3, 95% CI: 2.2–2.4) and 2.6-fold increased odds (AOR = 2.6, 95% CI: 2.3–3.0) of NHIS membership. Tertiary and high school graduates respectively had 2.4-fold- (AOR = 2.4, 95% CI: 2.0–2.9) and 1.4-fold increased odds (AOR = 1.4, 95% CI: 1.3–1.5) of being registered compared to individuals without any formal education. Urban residents were associated with 1.4-fold increased odds (AOR = 1.4, 95% CI: 1.3–1.5) of NHIS membership compared to their rural counterparts. Females were 1.4 times (AOR = 1.4, 95% CI: 1.3–1.5) more likely to be registered compared to males. The < 18-year-olds were 1.2 times (AOR = 1.2, 95% CI: 1.1–1.4) more likely to enroll compared to the elderly aged 70+ years. However, the working-age adults 18–59 year were 0.8 times (AOR = 0.80, 95% CI: 0.8–0.9) less likely to be registered compared to the elderly aged 70+ years. Individuals with no history of recent illness were 0.8 times (AOR = 0.8, 95% CI: 0.8–0.9) less likely to be registered compared to those who recently reported ill.

### Distribution of NHIS membership by category

Table 3 shows a summary of NHIS membership by category. The majority were from the informal sector (53.2%). This was followed by the exempt category comprising of the < 18-year-olds, elderly 70+ years and pregnant women (43.4%) and the formal sector (2.9%). The exempt (poor) recorded the lowest membership of 0.5%.

**Table 3:** Percentage distribution of NHIS membership by category in the Kassena-Nankana East and Kassena-Nankana West Districts in 2012. *N* = 19,641

Characteristics	Categories	Formal sector (*n* = 575)	Informal sector (*n* = 10,448)	Exempt (Poor) (*n* = 104)	Exempt (CEP) (*n* = 8514)	Total (*N* = 19,641)
Age (years)	< 18	1.0	19.9	0.4	78.6	8683
	18–59	4.8	85.9	0.6	8.7	8823
	60–69	3.6	61.7	0.4	34.4	1257
	70+	1.9	41.2	1.1	55.7	878
Gender	Female	2.2	56.6	0.6	40.6	10,872
	Male	3.8	49.0	0.5	46.7	8769
Location	Rural	2.1	51.8	0.6	45.5	16,386
	Urban	7.3	60.3	0.2	32.3	3255
Household size	1	13.7	60.5	0.9	25.0	468
	2–4	4.5	58.0	0.5	37.0	4618
	5+	2.1	51.5	0.5	46.0	14,555
Education*	No formal	1.2	66.3	0.6	31.9	4421
	Primary	1.2	47.4	0.4	51.0	5869
	High school	3.9	78.7	0.7	16.8	4183
	Tertiary	29.7	64.6	0.4	5.2	690
	Under schooling age*	0	24.3	0.5	75.3	4478
Ethnicity	Kassena	3.1	53.6	0.6	42.8	12,395
	Nankani	2.0	51.0	0.5	46.4	6086
	Others	6.2	60.2	0.2	33.5	1160
Religion	Christianity	3.8	54.6	0.5	41.1	11,626
	Traditional	1.4	49.4	0.6	48.6	6661
	Islam	3.3	59.2	0.1	37.4	1354
Wealth quintiles	Poorest	0.9	50.7	0.5	48.0	4626
	Moderately poor	1.5	50.6	0.8	47.1	3726
	Average	1.5	51.7	0.8	46.0	3444
	Moderately rich	2.1	55.9	0.5	41.5	4462
	Richest	9.8	57.4	0.2	32.6	3383
Recent illness	Yes	1.7	42.8	0.2	55.3	1156
	No	3.0	53.8	0.6	42.6	18,485
Overall (%)		2.9	53.2	0.5	43.4	100.0

Abbreviations: Exempt (CEP): Exempt (Children < 18, Elderly 70+ and Pregnant women)

In the informal sector, the highest proportions of NHIS membership were recorded among the working-age adults 18–59 years (85.9%), high school graduates (78.7%), single-member households (60.5%), urban residents (60.3%), Muslims (59.2%), members of the richest households (57.4%) and females (56.6%).

The highest proportions of NHIS membership in the formal sector were observed among the tertiary graduates (29.7%), single-member households (13.7%), the richest households (9.8%), urban residents (7.3%), adults of the working-age 18–59 years (4.8%) and males (3.8%).

The exempt CEP had the highest proportions in the < 18-year-olds (78.6%), those recently taken ill (55.3%), traditional worshippers (48.6%), the poorest households (48.0%), males (46.7%), five-member households (46.0%) and rural residents (45.5%). About one-fifth of the < 18-year-olds and over two-fifths of elderly 70+ years were found to have paid premium in order to register despite being exempt.

The highest proportions of enrollment for the exempt (poor) were recorded among the elderly 70+ years (1.1%), single-member households (0.9%), average or moderately poor households (0.8%), rural residents (0.6%) and females (0.6%). With respect to SES, the proportion of those exempt on the basis of poverty was slightly higher among the poorest households (0.5%) than their counterparts from the richest households (0.2%). When enrollment for the exempt poor and exempt CEP categories were combined, members from the poorest households had a higher proportion of 48.5% compared to 32.8% for those from the richest households. Of those exempts from premium payments either on accounts of poverty or other social vulnerability (*n* = 8618), only 1.2% were applied for the poor with members of the poorest households registering a slightly higher proportion than their counterparts from the richest households (1.0% vs. 0.7%).

### Reasons NHIS renewal by wealth quintiles

Willingness to renew membership was nearly universal for those who were currently registered (99.4%) with members from the poorest and richest households registering the same response rate of 99.3%.

Table 4 summarizes the reasons for individual willingness to renew membership. The most important reason was the ease with which they can access health care services (39.9%) and be guaranteed access without any financial burden in the event of ill-health (22.5%). Other respondents cited previous benefits received under the scheme (11.4%), good benefit package (8.3%) and premium being affordable (7.1%). There were marginal differences in these responses between the rich and poor except having access to care without any financial burden in which members of the poorest households registered 25.6% compared to 16.8% for those from the richest households. The following reasons were however rarely mentioned (< 1.0%) as the basis for renewing membership: good waiting time, good attitude of health workers and insurance staff, default SSNIT membership and exempt from premium payment.

**Table 4:** The reasons for NHIS registration by wealth quintiles. *N* = 19,513

Factors	Wealth quintiles					
	Poorest(*n* = 4592)	Mod poor (*n* = 3691)	Average (*n* = 3434)	Mod rich (*n* = 4436)	Richest (*n* = 3360)	Total (*N* = 19,513)
Premium affordable	6.2	6.1	8.9	8.5	5.7	7.1
Easy access to health care	41.3	40.5	40.9	37.6	39.0	39.9
Good benefit package	6.3	7.7	7.6	7.7	13.2	8.3
Guaranteed access to health care	25.6	24.4	21.6	22.7	16.8	22.5
Benefited from health insurance	10.7	11.9	9.6	12.5	12.1	11.4
Good attitude of insurance staff	0.3	0.0	0.2	0.5	0.1	0.2
Good quality of health care	1.5	1.4	1.3	1.9	1.0	1.4
Good waiting time	0.0	0.1	0.1	0	0	0.0
Drugs are available	1.7	2.3	2.3	2.5	1.2	2.0
Good attitude of health workers	0.2	0.0	0.1	0	0.1	0.1
Exempt from premium payment	0.1	0.2	0.4	0.4	3.7	0.9
Default SSNIT membership	0.1	0.1	0.1	0.1	1.5	0.3
Others	0.9	0.4	0.5	0.3	0.2	0.5
Don’t know	5.2	4.9	6.4	5.4	5.5	5.5

Abbreviations: SSNIT: Social Security and National Insurance Trust; Mod poor: Moderately poor; Mod rich: Moderately poor

### Barriers to NHIS registration and renewal by wealth quintiles

**Fig. 2:**
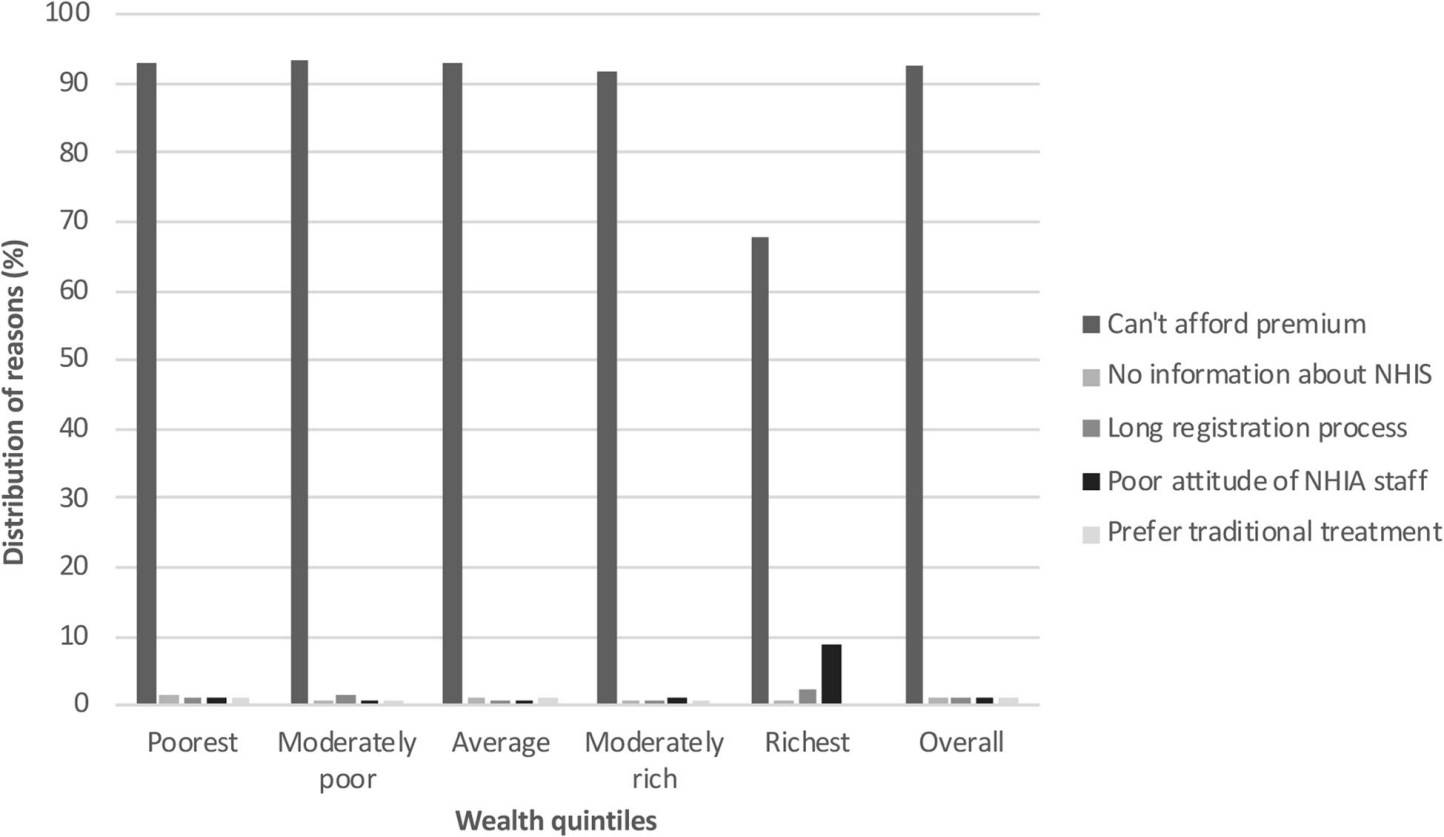
Top five barriers to NHIS renewal by wealth quintiles

Figure 2 presents a summary of the top five reasons for non-renewal across different socio-economic backgrounds. Inability to afford the insurance premium was the main reason why most previously registered members could not renew their membership (78.8%). This was more the case with members from the poorest households (80.0%) than their counterparts from the richest households (63.3%). The second reason was that many respondents were not aware their NHIS card had expired (8.4%); fewer from the poorest households than their counterparts from the richest households (7.5% vs. 17.1%). The long delay associated with the renewal process (4.4%) coupled with poor attitude of insurance staff (1.6%) were also turn-off to NHIS membership.

**Fig. 3:**
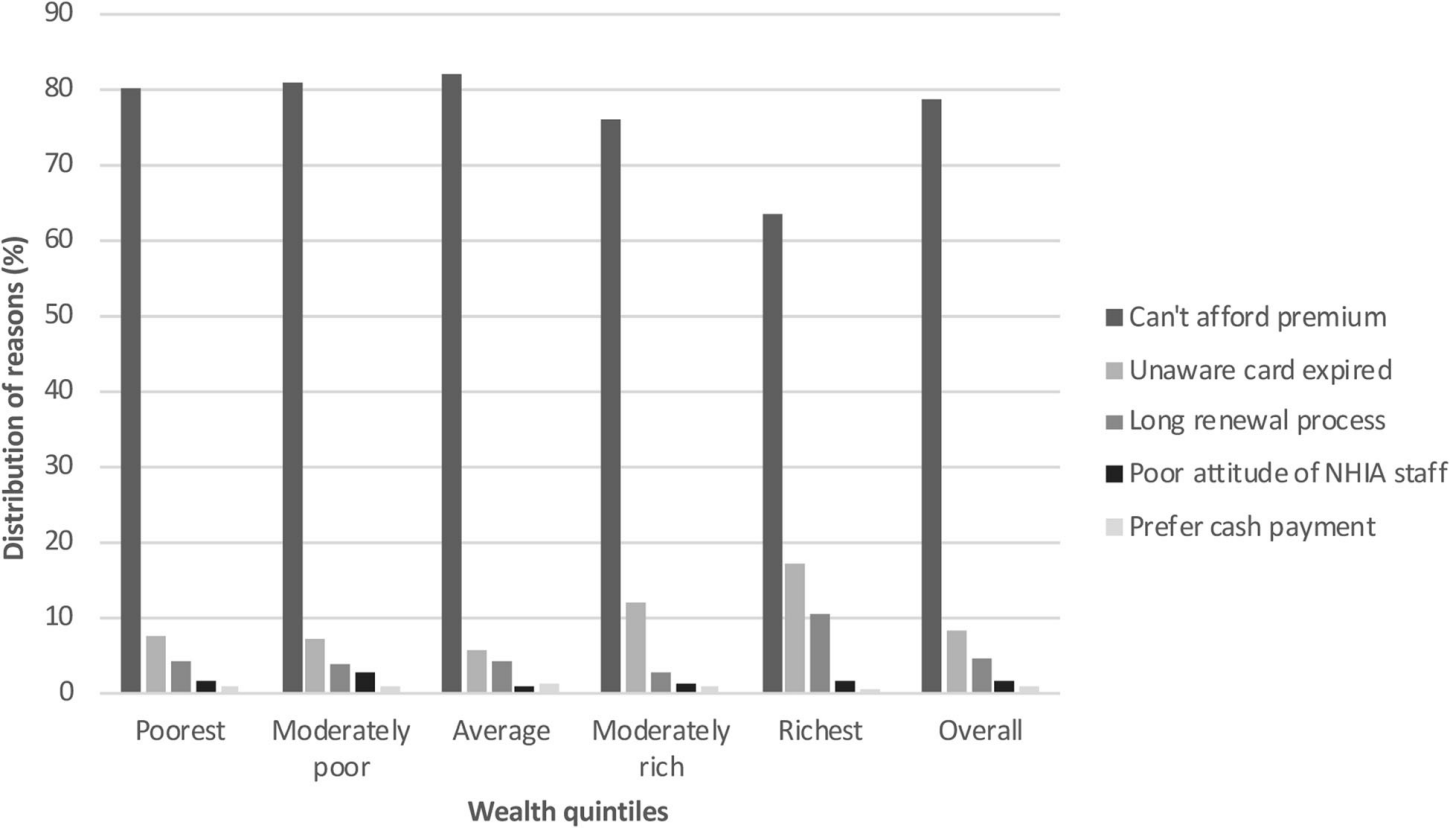
Top five barriers to NHIS registration by wealth quintiles

Figure 3 presents a summary of the top five reasons why some members have never registered with the scheme. Inability to afford insurance premium once again emerged as the main barrier to NHIS registration (92.6%). The response rate was generally high regardless of one’s socio-economic background. However, a much greater percentage of 92.7% was recorded among members from the poorest households compared to 67.7% from those of the richest households. About 1.1% of respondents indicated that they have never registered because they have no knowledge about the existence of the scheme. Again, long delay in processing NHIS card (1.0%) and poor attitude of insurance staff (1.0%) were the reasons some individuals have never joined the scheme. More members from the richest households than from the poorest households reported poor attitude of insurance staff as a turn-off to joining the scheme (8.7% vs. 1.1%).

## Discussion

Findings from our study show that only half of the population were registered with the scheme; the majority voluntarily paying for the cost of insurance. Members from the richest households, with higher education, living in an urban setting, females and children aged 18 years and below were among those significantly more likely to be registered with the scheme. Despite policy interventions aimed at ameliorating the poorest of the poor and vulnerable households against catastrophic health expenditure, only 0.5% of NHIS membership was on the basis of exemptions for the poor with slightly higher proportions applied for members of the poorest households than their counterparts from the richest households. The cost of premium was found to be the main barrier to NHIS membership with the poorest households being worst affected.

Firstly, our findings demonstrate that NHIS coverage at the two adjoining districts of Kassena-Nankana in 2012 was higher than the national coverage of 36% and in earlier survey in a rural district of the Ashanti Region in 2008 that recorded 38% [16, 26]. Similarly when compared with a 2010/2011 survey based on validly seen NHIS cards at seven other districts of the Upper East Region among women of reproductive, our observed coverage was higher by 10% [27]. The relatively higher enrollment in the Kassena-Nankana districts may be attributable to the operations of the NHRC through its implementation of high-quality health research and social programs for the resident population. The implementation of these programs is often accompanied with substantial investments for improving hospital diagnostic capacities, health worker performance and free registration of insurance for clinical trial participants who may need to access routine medical care services during trials. Such generated benefits to local health systems reported at most sentinel sites in Africa where improved standards are pre-requisites for the conduct of clinical trials [28–30] may have facilitated the higher NHIS enrollment in the study area.

Secondly, our findings nonetheless highlight great disparities in NHIS enrollment against members of the poorest households, those without formal education and living in rural areas. This finding is similar to observations by Akazili et al. in seven other districts of the Upper East region that demonstrated that women of lower SES, living in rural settings and have no formal education were less likely to register with the scheme [27]. The inadequacy of health care facilities within reasonable reach of rural residents requires that they travel longer distances at greater cost to access health care services unlike their urban counterparts [16]. The associated high non-medical cost is a disincentive for rural residents and poorer households to purchase insurance [31, 32]. This may explain the observed lower levels of registration among members of the poorest households and rural residents in the Asante Akyem study that showed significant association between NHIS registration and distance to a health care provider with those living farthest less likely to register. Given the high tax component of the funding mix of the scheme, there are equity concerns as poorer households mostly from rural communities with lower purchasing power invariably cross-subsidize the cost of health services for urban residents. The siting of more smaller health facilities such as community health planning services in underserved communities will improve geographical access to health services and thereby encourage the poor and rural residents to enroll [33].

Thirdly, we found that exemptions for the vulnerable groups (< 18-year-olds, elderly aged 70+ years and pregnant women) accounted for 43.4% compared to 0.5% for exempt poor with the latter constituting only 1.2% of total exemptions applied either on accounts of poverty or other social vulnerabilities. The increased likelihood of enrollment among the < 18-year-olds and elderly aged 70+ years relative to working-age adults 18–59 years suggests that mechanisms for identifying beneficiaries based on age were more efficient. Notwithstanding, it was observed that about 20% of the < 18-year-olds and over 40% of the elderly who were supposed to enjoy exemption paid premium in order to register. This observation is consistent with findings from a study examining the implementation of the exemption policy in the three ecological zones of Ghana that found premium payment by exempt groups still exist especially among children and the elderly 70 years and above [34]. Allowing the exempt category to pay premium is in contravention of NHIA regulations and may likely be the basis of the reported poor attitude of insurance staff in our survey.

Exemption policies have mostly been implemented to the disadvantage of the poor. Available records predating the introduction of the NHIS at a district hospital in the Upper West Region from 2001 show a inexplicably low proportion of 0.4% of the poor being granted exemption which only increased marginally to 1% in 2004 compared to children five years and below which ranges from 23 to 29% and pregnant women from 24 to 53% [35]. It is evident that a more targeted mechanism is required to ensure equitable representation of the poor. However, the current modality for identifying the poor by the Department of Social Welfare (DSW) through the Livelihood Empowerment Against Poverty (LEAP) program holds little hope of reversing this inequity. The program which has the goal of ameliorating short-term poverty to encourage long term human development by identifying extremely poor households for cash and health insurance benefits is bedeviled with many challenges [36, 37]. The criteria based on a lack of income due to unemployment and shelter or having no support from any other persons have been proven to be inconsistent with the widely considered view of poverty in the community [38]. Little is known about the program among the poor due to inadequate publicity by the DSW [39]. Moreover, administrative difficulties, inadequate funding and perceived political inferences undermine the operations of the LEAP program.

Formal sector workers are exempt from premium payment but required to pay processing fee for NHIS at the point of registration due to the funding architecture of the scheme which has a 2.5% of workers’ SSNIT pension fund deducted at source. Enrollment of the formal sector was therefore expected to be high. However, the relatively low formal sector membership of 2.9% in predominantly rural setting where the majority are farmers is not unusual. Further research is recommended to explain if the low enrollment of formal sector workers is consistent with the observed declining national rates from 4.7% in 2010 to 3.3% in 2015 [40].

Fourthly, we also found that cost of premium was the main barrier to NHIS registration and renewal with poorer households being worse affected. Our finding is consistent with existing literature in most LMIC including Ghana that shows affordability of premium as a challenge among the individual with the lowest SES [41–44]. The lower enrollment among the poorest households can be explained by the fact that poorer households have little disposable income and are therefore less likely to sacrifice their immediate basic needs of life such as food, shelter and clothing at the expense of purchasing insurance for future health needs. Other barriers to enrollment were inadequate information about the scheme, poor attitude of insurance staff and long delay in processing NHIS cards. Our study confirms reports of Upper East as the region with the worst delay in processing of NHIS cards in a national survey involving 145 DHMIS in 2011 [45]. This is particularly discouraging and may render enrollment onto the scheme unattractive for new members. The introduction of instant NHIS cards at the point of registration and electronic renewal platform are likely to alleviate the protracted registration and renewal process; and encourage subscription to the scheme [46, 47]. The high mobile phone penetration in Ghana presents a unique opportunity for NHIA to explore the use of voice or short messaging services as tools to educate the general public about the operations of the scheme. This can include sending reminders to clients whose memberships are nearing expiration to avoid inadvertent loss of membership. With respect to the factors influencing the decision to register and retain membership, the general recognition that being insured makes access to health care services easy with the guarantee of being financially protected is indicative of the ability of the scheme to offer some relief against catastrophic health care expenditures. The decision to retain membership due to previous benefits as clients across the different socio-economic background confirms this assertion. Our result further adds to the growing literature that convenience of access to care and financial protection in the event of illness are among the strongest predictors of enrollment and retention decisions associated with the Ghana NHIS [44, 48, 49].

Ghana has achieved considerable success by designing a scheme that covers different segments of the population including the rich, poor and workers from both the formal and informal sectors. However, effective and more targeted mechanisms to provide adequate cover for poor households are still required. Standardized longitudinal data of NHIS coverage for the resident population of Ghana from 2010 show an initial increase from 33 to 41% in 2015 but declined to 35% in 2017 [40]. Although, NHIS enrollment in the two adjoining districts of Kassena-Nankana are higher than the national coverage, approximately half of the resident population are uninsured, more efforts are required by the NHIA to ensure adequate enrollment for the entire Ghanaian population. Progress made by Rwanda, a LMIC in attaining national health insurance coverage of 85% after eight years of implementation demonstrates that UHC is an achievable goal [50]. The Rwanda approach which involved the use of community-based insurance schemes targeted at the rural poor backed with intensive community mobilization presents as an alternative that many LMIC, including Ghana can consider.

### Limitations

We urge the interpretation of our findings in the context of the following acknowledged limitations. The proportion of individuals insured on the NHIS is likely to be underestimated. This is due to the exclusion from the analysis of individuals who reported to be insured but their NHIS cards could not be verified by the field data collectors. The use of heads of household as proxy respondents may introduce some biases, however these biases/errors are most likely to be minimal given the fact that heads of household are the custodians of important family documents including NHIS cards and are more likely to know the insurance status of all members of their households.

## Conclusion

Findings from our study show that only half of the resident population in the two districts of Kassena-Nankana were registered with the NHIS. Despite the implementation of as exemption policy as an equity measure, members of the poorest SES were rarely identified for exemptions due to lack of effective targeting mechanism. The government must resource the DSW to adopt community-centered approach based on local indicators of poverty to identify the poorest of the for exemptions.

## Data Availability

The datasets generated during and/or during the current study are not publicly available. The datasets are privately owned by the Navrongo Health Research Center, Navrongo, Ghana, but are available from the corresponding author on reasonable request.

## Abbreviations

AIDS: Acquired Immune Deficiency Syndrome
AOR: Adjusted odds ratio
CHPS: Community-based Health Planning Services
DMHIS: District-wide Mutual Health Insurance Schemes
DSW: Department of Social Welfare; Exempt CEP: Exempt (Children, Elderly and Pregnant women)
GH¢: Ghana cedi
GLSS: Ghana Living Standard Survey GOG: Government of Ghana
GSS: Ghana Statistical Service
HIV: Human immuno-deficiency virus
LEAP: Livelihood Empowerment Against Poverty
LMIC: Low and Middle-Income Country
NHDSS: Navrongo Health and Demographic Surveillance System; NHIA: National Health Insurance Authority NHIS: National Health Insurance Scheme
NHRC: Navrongo Health Research Center
Perm ID: Permanent Identification
SES: Socio-economic status; SSNIT: Social Security and National Insurance Trust
SGD: Sustainable development goals
UHC: Universal health coverage
VAT: Value added tax
WHO: World Health Organization
